# A unifying framework for fast randomization of ecological networks with fixed (node) degrees

**DOI:** 10.1016/j.mex.2018.06.018

**Published:** 2018-07-05

**Authors:** Corrie Jacobien Carstens, Annabell Berger, Giovanni Strona

**Affiliations:** a University of Amsterdam, KdV Institute for Mathematics, Amsterdam, Netherlands; b Martin Luther University Halle-Wittenberg, Institute of Computer Science, Halle(Saale), Germany; c European Commission Joint Research Centre, Directorate D – Sustainable Resources, Bio-Economy Unit, Ispra (VA), Italy

**Keywords:** Curveball algorithms to randomize networks with fixed node degrees, Binary matrices, Co-occurrence, Curveball algorithm, Food web, Null model

## Abstract

The Curveball algorithm is an efficient and unbiased procedure for randomizing bipartite networks (or their matrix counterpart) while preserving node degrees. Here we introduce two extensions of the procedure, making it capable to randomize also unimode directed and undirected networks. We provide formal mathematical proofs that the two extensions, as the original Curveball, are fast and unbiased (i.e. they sample uniformly from the universe of possible network configurations).

• We extend the Curveball algorithm to unimode directed and undirected networks.
• As the original Curveball, extensions are fast and unbiased.
• We provide Python and R code implementing the new procedures.

## Graphical abstract

**Figure:**
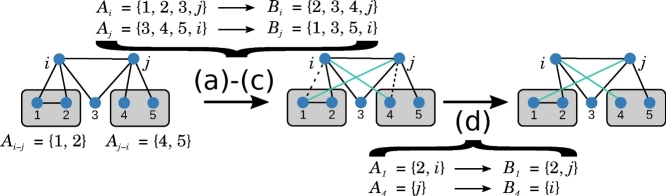

**Specification table**

**Table:** 

Subject Area	*Agricultural and Biological Sciences*
More specific subject area:	*Network science*
Method name:	*Curveball algorithms to randomize networks with fixed node degrees*
Name and reference of original method	*Curveball algorithm: G. Strona, D. Nappo, F. Boccacci, S. Fattorini, J. San-Miguel-Ayanz. A fast and unbiased procedure to randomize ecological binary matrices with fixed row and column totals. Nat. Commun. 5 (2014) 4114.*
Resource availability	*https://github.com/queenBNE/Curveball*

## Methods details

### Background

A good algorithm to generate random networks with prescribed degree distribution (which is identical to the issue of generating random binary matrices with fixed marginal totals) should have two properties: it should be able to generate any one among all possible networks having a certain node degrees with the same probability, i.e. it should not tend towards the generation of networks having particular structural properties; and it should be able to generate truly random networks fast.

Markov chains, where the randomization takes place in subsequent steps, each involving a small change in the network structure, represent a common solution to this problem. Several network randomization Markov chains have been shown to converge to the uniform distribution on their state space, that is they have been shown able to generate truly random networks with prescribed degree distribution [1], [2], [3], [4], [5]. By contrast, most of Markov chains exhibit an important limit, that is it is not clear how many randomization steps they require to ensure that the randomized network is truly random.

The best known Markov chain approach for randomizing network while preserving their degree sequence is the *switching model* (also known as rewiring, switching chain and swapping edges) [6], [7], [2], [8]. It can be applied to different kinds of networks, being able to properly randomize bipartite networks, undirected networks or directed networks with given node degrees, by repeatedly switching the ends of non-adjacent edge pairs (with some additional rules required for the correct randomization of directed networks, [2]). Yet, this method has a fundamental drawback, which is requiring a very large number of switches in order to ensure an unbiased randomization, which grows very rapidly with the size of the network (see, for example, [9]).

A more recent Markov chain approach is the Curveball algorithm [10]. Experimentally, this chain has been shown to mix much faster than the corresponding switching chain [10]. Why the Curveball algorithm mixes faster than the switching model can be understood when thinking of both algorithms as games in which kids trade cards. That is, think of the Curveball algorithm as an algorithm that randomises the binary *n* × *m* bi-adjacency matrix of a bipartite network. Imagine that each row of the adjacency matrix corresponds to a kid, and the 1's in each row correspond to the cards owned by the kid. Then at each step in the Curveball algorithm, two kids are randomly selected, and trade a number of their differing cards. Using this same analogy for the switching model, in each step two cards are randomly selected and traded if firstly they are different and secondly they are owned by different kids (note that various algorithms implementing similar approaches were discovered independently by Verhelst [4]). Intuitively, the Curveball algorithm is clearly a more efficient approach to randomise the card ownership by the kids. More formally, the Curveball algorithm is also based on switches but instead of making one switch, several switches can be made in a single step, which leads to possibly exponentially many networks being reached in a single step, in contrast with the switching model where at most *n*^4^ (the maximum number of possible edge pairs) networks can be reached in a single step.

Designed to randomize only bipartite networks, the Curveball permits the randomization of both species × locality matrices, and bipartite ecological networks such as host-parasite and plant-pollinator ones. There is, however, an important reason why such design requires an urgent upgrade. Notably, bipartite ecological networks have often been studied separately from food webs, even though all those networks belong to the same broader ecological class of ‘resource-consumer’ networks [11].

Food webs belong to a different class of networks, that is directed networks. In such networks, nodes cannot be attributed unambiguously to two different classes, since the same node can be simultaneously a consumer and a resource (for example, a predator can be eaten by another predator of a higher trophic level) [12]. A third class of networks is that of undirected networks, which has importance in various fields, such as social sciences and epidemiology, with networks of those kinds being well suited, to represent contacts between persons, and that is also becoming increasingly relevant in the ecological context. In fact, there is a growing interest in the study of co-occurrence networks, that is networks obtained by linking species found together more often than random expectation, and hence considered as potentially interacting (see, for example, [13], [14], [15]).

Although some attempts has been recently made to provide measures of network structure applicable to different kinds of ecological networks (see, for example, [16], [17]), we are still very far from having a unifying analytical toolbox. Here we take a step further in this direction, by showing how the efficient Curveball algorithm can be extended to work also with unimode directed networks, and undirected networks. Besides providing ecologists with a common procedure to analyze different ecological entities, this constitutes an important advance for network science in general, with the potential of bringing benefit to various disciplines.

### Extending the Curveball algorithm

We now propose two extensions of the Curveball algorithm: the Directed Curveball algorithm, which samples directed networks and the Undirected Curveball algorithm, which samples networks (Throughout this paper we use the convention that both directed and undirected networks do not contain self-loops or multiple edges). Both are Markov chain algorithms, which randomise networks by repeatedly applying them small changes, in this case trades of elements.

### The Directed Curveball algorithm

An efficient way to store a directed network *G* = (*V*, *E*) is by storing its adjacency list, which is also the most natural data structure to run the Directed Curveball algorithm on. The adjacency list of *G* is a list of sets $A_{v}$, one for each node v∈V, where the set $A_{v}$ contains all out-neighbours of v.

The Directed Curveball algorithm randomises a directed network by repeatedly applying *trades* to its adjacency list. A *trade* is defined as follows: (a) select two sets *A*_*i*_ and *A*_*j*_ at random, (b) Let *A*_*i*−*j*_ be all nodes in *A*_*i*_ that are not in *A*_*j*_ and that are *not equal to j*, i.e. *A*_*i*−*j*_ = *A*_*i*_ ∖ (*A*_*j*_ ∪ {*j*}) and similarly let *A*_*j*−*i*_ = *A*_*j*_ ∖ (*A*_*i*_ ∪ {*i*}). (c) Create new sets *B*_*i*_ by removing *A*_*i*−*j*_ from *A*_*i*_ and adding the same number of elements randomly chosen from *A*_*i*−*j*_ ∪ *A*_*j*−*i*_. Combine *A*_*j*_  *A*_*j*−*i*_ with the remaining elements of *A*_*i*−*j*_ ∪ *A*_*j*−*i*_ to form *B*_*j*_.

Notice that the definition of *A*_*i*−*j*_ = *A*_*i*_ ∖ (*A*_*j*_ ∪ {*j*}) in step (*b*) ensures no self-loop can be created at node *j*, since this would require adding *j* to *A*_*j*−*i*_.

**Fig. 1:**
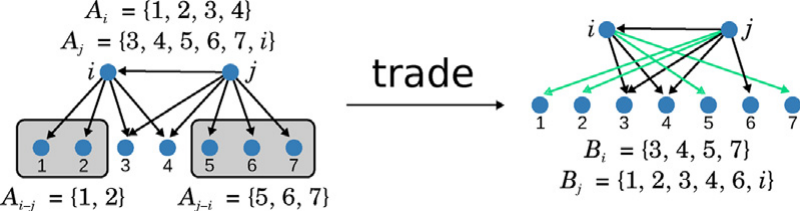
Illustration of a trade in the Directed Curveball algorithm. Vertex *i* and *j* have several common out-neighbours and *i* is an out-neighbour of *j*. Those are removed to obtain the set {1, 2, 5, 6, 7} of nodes that can be traded. The nodes 5 and 7 are selected as new neighbours for *i*, the trade results in the network on the right.

Fig. 1 illustrates a trade of the Directed Curveball algorithm. We will refer to the number of elements exchanged as the *size of a trade*, for instance the trade in Fig. 1 is of size two. It is possible for a trade to be of size zero, in this case the current network is repeated and we move on to the next trade. The Lemma below shows that all switches in the switching model for directed equal trades of size one in the Directed Curveball algorithm. But, the Directed Curveball algorithm in addition allows trades of larger size. Intuitively, this could reduce the number of steps needed to obtain a random sample as compared to switching chains, since a trade can introduce more randomness than a switch.

Lemma 1 *Any switch in a directed network is a trade of size one of the Directed Curveball algorithm.*

Proof Let (*x*, *y*) and (u,v) be edges in a directed network *G* that are allowed to be switched. Then x≠v and *u* ≠ *y* since otherwise this switch would introduce a self-loop. Furthermore $v\notin A_{x}$ and *y* ∉ *A*_*u*_ since otherwise the resulting directed network would have multiple edges. In particular this implies *y* ∈ *A*_*x*−*u*_ and $v\in A_{u-x}$. Now if row *x* and row *u* are selected for a trade, then $B_{x}=(A_{x}.\{y\})\cup \{v\}$ and $B_{u}=(A_{u}.\{v\})\cup y$ are possible sets in step (c) that lead to exactly the two new edges (x,v) and (*u*, *y*).

In the Appendix we show that the Directed Curveball algorithm converges to the uniform distribution whenever the switching chain for directed networks does. That is, eventually after a large number of trades, we obtain a network sampled from the uniform distribution. Note that, for certain node degrees, not all networks realisations (i.e. networks with the given node degrees -) can be obtained by applying switches to a given network.

The simplest example is the oriented triangle (1, 2), (2, 3), (3, 1). No swap or trade can be applied, and hence we can never generate the triangle (1, 3), (3, 2), (2, 1), which is also a possible network. Adding a second procedure, i.e. reorienting a randomly chosen directed triangles (‘hexagonal move’) solves this problem completely [2]. However, further work showed that not all of these triangles need to be re-oriented [18]. Theorem B3 in Appendix can be used to create a fast algorithm to recognize the triangles which need to be re-oriented in a network, and to choose one random orientation for each of them. Using the Curveball chain after this step delivers a uniform randomly sampled network. Furthermore, depending on the purpose of the randomization, this step might be even not necessary.

Berger et al. [18] proved that (in cases where only network topology matters, i.e. where the information about the identity of individual nodes can be discarded) the sole use of the switch chain permits to sample uniformly at random from the set of all possible networks (which is a subset of the full set of networks including those that could be generated by triangle re-orientation). This would be enough to compare the frequency of network structural patterns such as motifs, nestedness, or C-score between empirical and randomized networks, since their proportion is not affected by the re-orientation step. Conversely, the re-orientation step discussed in Appendix would be necessary to assess the significance of patterns affected by the identity of nodes (for example, the frequency of a specific directed edge (*i*, *j*) in all networks).

### The Undirected Curveball algorithm

The Undirected Curveball algorithm samples networks with fixed node degrees. The adjacency list representation of a network *G* = (*V*, *E*) is a list of sets *A*_*i*_. The set *A*_*i*_ contains the indices of the neighbours of vertex *i*. For undirected networks each edge {*i*, *j*} is represented twice in the adjacency list, since *i* ∈ *A*_*j*_ and *j* ∈ *A*_*i*_. Furthermore, *i* ∉ *A*_*i*_ for all *i*, since networks do not contain self-loops.

A trade in the Undirected Curveball algorithm is defined by the following steps. (a) Randomly select two sets *A*_*i*_ and *A*_*j*_. (b) Let *A*_*i*−*j*_ be the set of elements in *A*_*i*_ not in *A*_*j*_ and not equal to *j*, i.e. *A*_*i*−*j*_ : = *A*_*i*_  (*A*_*j*_ ∪ {*j*}). Analogously define *A*_*j*−*i*_ : = *A*_*j*_  (*A*_*i*_ ∪ {*i*}). (c) Create a new set *B*_*i*_ by removing *A*_*i*−*j*_ from *A*_*i*_ and adding the same number of elements randomly chosen from *A*_*i*−*j*_ ∪ *A*_*j*−*i*_. Combine *A*_*j*_  *A*_*j*−*i*_ with the remaining elements of *A*_*i*−*j*_ ∪ *A*_*j*−*i*_ to form *B*_*j*_. (d) For each node *k* ∈ *B*_*i*_  *A*_*i*_, replace *j* by *i* in *B*_*k*_, similarly for each *l* ∈ *B*_*j*_  *A*_*j*_, replace *i* by *j* in *B*_*l*_.

**Fig. 2:**
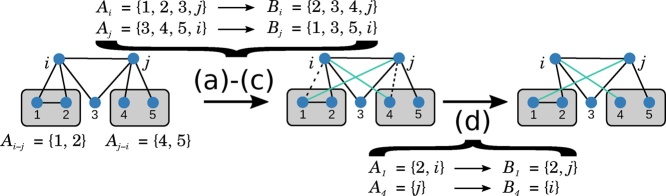
Illustration of a trade in the Undirected Curveball algorithm. Nodes *i* and *j* are neighbours and have a single common neighbour. The nodes that are available for trading are nodes 1, 2, 4 and 5. A trade of size one is performed, exchanging nodes 1 and 4, hence in step (d) the sets *A*_1_ and *A*_4_ are updated.

Step (b) ensures no self-loops are introduced and step (d) ensures that *B* represents a network (i.e. that *i* ∈ *B*_*j*_ implies *j* ∈ *A*_*i*_). Fig. 2 illustrates a trade in the Undirected Curveball algorithm. Note that the same trade can be made in the opposite direction, i.e. *B* network can be transformed into *A* in a single trade. A proof for this (which is a necessary condition for an unbiased Markov chain) is provided in Appendix.

**Fig. 3:**
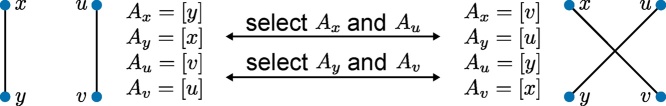
A switch corresponds to a trade of size one in the Undirected Curveball algorithm. Notice that each switch can be realized by two distinct trades: the switch from {*x*, *y*} and {u,v} to {x,v} and {*u*, *y*} can be realized by selecting *A*_*x*_ and *A*_*u*_ and trading *y* for v*or* by selecting *A*_*y*_ and $A_{v}$ and trading *x* for *u*.

The Undirected Curveball algorithm includes trades of size zero which correspond to repeating the current network. Furthermore, Lemma 2 shows that any switch in the switching model for corresponds to a trade of size one in the Undirected Curveball algorithm. In fact, Fig. 3 shows that for each switch in the switching model, there are two different trades of size one in the Undirected Curveball algorithm.

Lemma 2 *Let G*, *G*′ *be that differ by a switch. There are two trades of size one in the Undirected Curveball algorithm from G to G*′*.*

Proof Without loss of generality we may assume that *G* = (*V*, *E*) and *G*′ = (*V*, *E*′) differ by a switch from {*x*, *y*} and {u,v} to {x,v} and {*u*, *y*}. Let {*A*_1_, …, *A*_*n*_} be the adjacency set representation of *G*, then *y* ∈ *A*_*x*−*u*_ since the edge {*x*, *y*} is an edge of *G*, the edge {*u*, *y*} is not and *y* can not be equal to *u* since {*u*, *y*} ∈ *E*′ and *G*′ has no self-loops. Similarly we find that $v\in A_{u-x}$ and hence the trade that swaps *y* and v between rows *x* and *u* results in the network *G*′. Similarly, there is a second trade which generates *G*′, namely the trade that exchanges *x* and *u* between sets *A*_*y*_ and $A_{v}$. □

Analogous to the other versions of the Curveball algorithm, the Undirected Curveball algorithm in addition allows trades of larger size, corresponding to making several switches at once. In the Appendix we prove that the Undirected Curveball algorithm samples uniformly at random after applying a large number of trades.

### Mixing time and experimental stopping times

There are some obvious major differences between the typical implementation of switch-based algorithm and that of Curveball algorithms that stem from the use of the adjacency list representation of a network used by the latter, compared to the edge-list representation used by the first. For example, the most common procedure used for fixed-degree network randomization [19] consists, at each step, in sampling two pair of linked nodes, a–b and c–d and rewire them in the form a–d and c–b if and only if neither a–d nor c–b exist already in the network. It is clear that this procedure has two important limitations in terms of performance, one deriving from the probability of performing a successful swap, and the other one deriving from the need to check that performing a given swap will not result in the generation of multiple edges. While the first limitation is strongly dependent on network structure (and we may also imagine situations where the probability of performing a successful swap is identical to the probability of performing a successful Curveball trade), the additional check for multiple edges is a serious bottleneck limiting the efficiency of swap-based algorithms (and one narrowing with network size). As a consequence, performing a performance comparison (for example in terms of CPU time needed to properly randomize a network) between typical swap implementations and their Curveball counterparts would be a trivial exercise. However, the most important question for practitioners as well as theoreticians is how many steps the Curveball algorithms have to run from an initial probability distribution (where an initial state is taken from) to sample from a probability distribution which is close to the uniform distribution. This number is defined as the *total mixing time*, i.e. the number *N* of reiteration steps in the Curveball algorithms.

The Curveball algorithm has experimentally been shown to run much faster than the switching algorithm [10]. Intuitively, this property should extend also to the directed and undirected versions of the Curveball. We tested this experimentally, by comparing the total mixing time of the Curveball algorithm with that of the switching model in: two sets of random (i.e. Erdős-Rényi) networks including, respectively, 100 directed and 100 undirected networks having a number of nodes (*V*) extracted at random between 100 and 1000, and a number of edges equal to *E* × *V*, with edges varying randomly between 5 and 50; two sets of power law (i.e. Albert Barabási) networks including, respectively, 100 directed and 100 undirected networks having a number of nodes (*V*) extracted at random between 100 and 1000; and two empirical networks, namely a directed food-web (listing all trophic interactions recorded in Little Rock Lake, Wisconsin in the United States of America, [20], having 183 nodes and 2476 edges), and an undirected co-occurrence network (representing ecological interactions between bacteria [21], having 316 nodes and 1086 edges).

In order to compare the asymptotic mixing times of these algorithms, that is to assess the increased performance stemming from trading multiple elements at once compared to performing individual swaps, while excluding the above mentioned limitations emerging from the different algorithms’ design, we implemented a swap algorithm in the same form of the Curveball but with the additional constrain of permitting only trades of size 1 between adjacency lists.

To track the two algorithms’ convergence towards the uniform distribution, we used, as a proxy, the degree of network perturbation, that we measured as the fraction of edges in the target network differing from those in its randomized counterpart [10]). For both the Curveball and the (modified) swap procedure, we recorded network perturbation every 100 step (i.e. trades and swaps), performing a total of 25,000 steps.

**Fig. 4:**
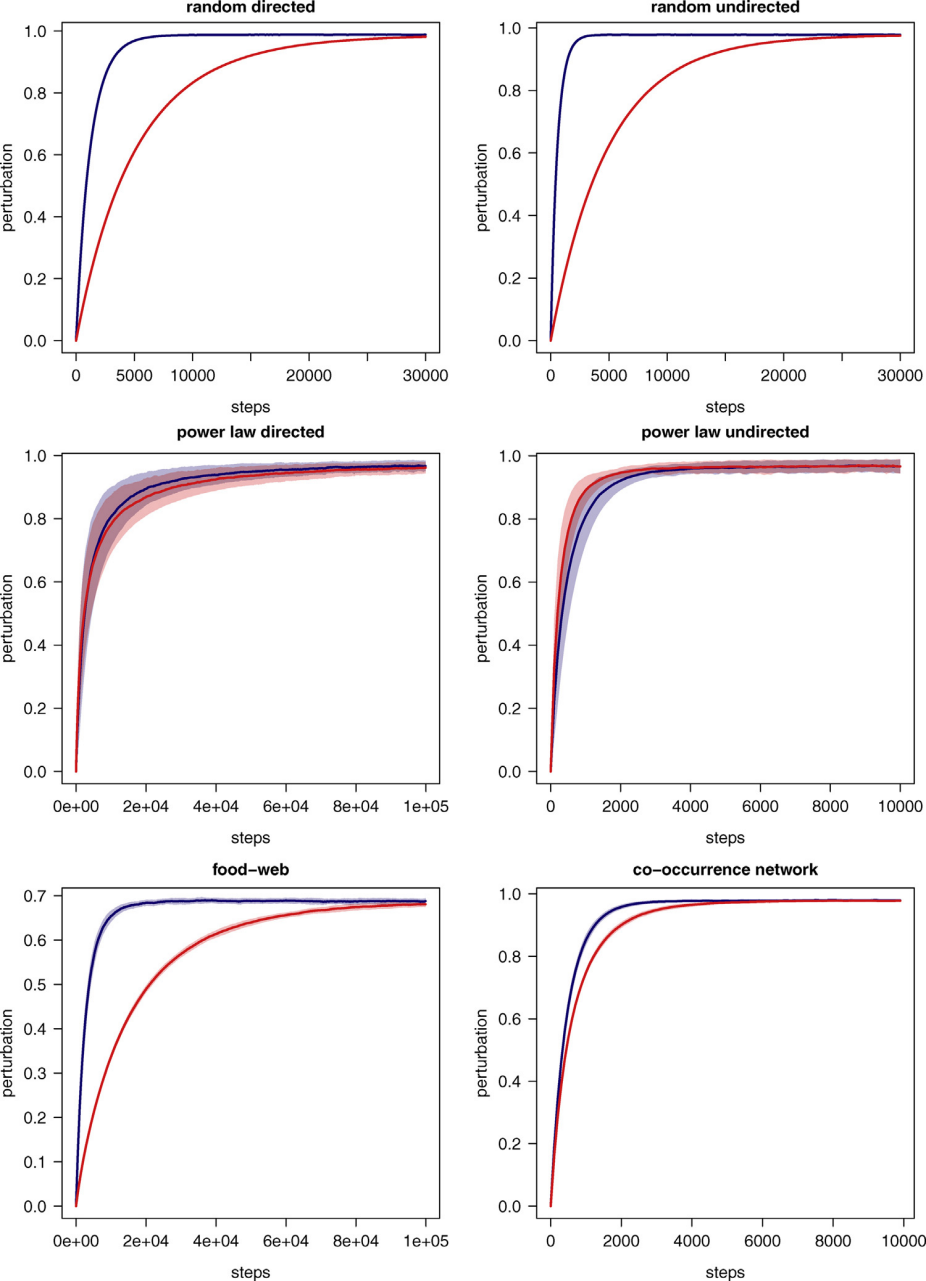
The degree of network perturbation (measured as the fraction of edges in a randomized network differing from those of the original network) for an increasing number of steps in the Markov chains of the modified swap procedures (red), and of the Curveball algorithms (blue) while randomising: a set of one hundred random (Erdős-Rényi) directed; a set of one hundred random (Erdős-Rényi) undirected; a food-web; and a co-occurrence network. The randomization of the food-web and of the co-occurrence network was replicated one hundred times. Solid lines indicate the average values over the replicates, while shaded areas indicate standard deviation.

In Fig. 4 we show how the Curveball algorithms converge much faster than the switching chain in both the random (Erdős-Rényi) and real world networks. The improvement of the Curveball algorithms against the switching chain was less pronounced for the power law (Albert Barabási) networks. This can be explained by the fact that all vertices in this network have low out-degree (1, 2 or 3), and hence the number of elements traded by the Curveball at each step is often one (i.e. equivalent to a step in the swap procedure). Furthermore, due to the power-law degree distribution, many of the edges will have the same target further limiting the size of trades.

All algorithms were implemented in the R and Python programming language. All the code using in our analyses is publicly available [22]. We also provide user friendly functions implementing the new algorithms in both Python and R programming language as Supplementary material.

### Concluding remarks

The increasing understanding of natural systems’ complexity is making clear how the current separation between food-web science, and mutualistic and antagonistic bipartite ecological networks may hide important patterns and processes possibly responsible for the emergence and maintaining of diversity [23,24]. New analytical tools are needed to overcome this issue, and there is still long way towards a truly organic theory of ecological interactions. By providing a unifying framework for the randomization of all kinds of networks relevant to the ecological field, we hope that this work may represent a further step in that direction.

Finally, we have focussed here mainly on ecological networks as this was the main motivation behind the development of the original Curveball, and behind our interest in extending its application beyond bipartite networks. Nevertheless, the randomization of large directed and undirected networks is a compelling problem in several fields other than ecology. For example, investigating the structure of social networks is becoming more and more important to improve our understanding of complex societal mechanisms [25], and disease spread dynamics [26]. We are confident that our new methods will be useful in those fields too.

## Appendix A Supplementary data

Mathematical proofs
