# Supplementary material for: A unifying framework for fast randomization of ecological networks with fixed (node) degrees
Source: MethodsX. 2018 Jul 5;5:773–80. doi: 10.1016/j.mex.2018.06.018 (PMC6072652; doi:10.1016/j.mex.2018.06.018)
Supplement: Supplementary file 1 [file mmc1.pdf]

Supplementary Information for: A unifying framework for fast randomization of ecological networks with fixed (node) degrees

Corrie Jacobien Carstens

University of Amsterdam, KdV Institute for Mathematics, Amsterdam, Netherlands

Annabell Berger

Martin Luther University Halle-Wittenberg, Institute of Computer Science, Halle(Saale), Germany

Giovanni Strona

European Commission Joint Research Centre, Directorate D - Sustainable Resources, Bio-Economy Unit, Ispra (VA), Italy

AppendixA. Convergence of Markov Chains

A Markov chain can be seen as a *random walk* [1] on a set Ω of combinatorial objects, the so-called *states*. Two states $x, y \in \Omega$ are connected via a transition edge $(x, y) \in \Psi$, when x can be transformed into y via a small local change. For the switching chain such a 'local change' corresponds to a switch, and in the Curveball algorithms to one trade. For both algorithms, the states of Ω are all realisations of a fixed degree sequence. This definition induces a so-called directed *state graph* $\Gamma = (\Omega, \Psi)$, representing the states and how they are connected by local changes. A step from x to y in a random walk is done with *transition probability* p_{xy}.

In [2] the Curveball algorithm was proven to converge to the uniform distribution by applying the fundamental theorem for Markov chains (see for example [3]).

Theorem AppendixA.1. *A finite Markov chain converges to its unique stationary distribution if its state graph $\Gamma = (\Omega, \Psi)$ is connected and non-bipartite.*

If there exists a probability distribution $\pi : \Omega \mapsto [0,1]$ *such that the detailed balanced equations,* $\pi(x)p_{xy} = \pi(y)p_{yx}$*, are satisfied for all* $(x,y) \in \Psi$*, then* π *is this unique stationary distribution.*

This theorem implies that Markov chains which fulfil these properties converges to the uniform distribution if $p_{xy} = p_{yx}$ for all $x, y \in \Omega$.

We now derive the conditions for which the Directed Curveball algorithm, the Undirected Curveball algorithm converge to the uniform distribution on their respective state spaces.

Definition AppendixA.2. *Let* X *and* Y *be disjoint sets. The elements* $X \cup Y$ *can be divided into two sets of sizes* $|X|$ *and* $|Y|$ *in* $\binom{|X|+|Y|}{|X|}$ *different ways. We define* p_{ij} *as* $\binom{|X|+|Y|}{|X|}^{-1}$*, the probability of choosing one such set uniformly at random, when* $X = A_{i-j}$ *and* $Y = A_{j-i}$*. This is exactly the probability of choosing a specific trade between rows* i *and* j*.*

AppendixA.1. Uniform sampling Undirected Curveball

To determine the transition probabilities of the Undirected Curveball algorithm we need to distinguish between two cases. Firstly, when A and B differ in trade of size larger than one, there is a unique pair $\{i, j\}$ of rows that corresponds to this trade, and the transition probability is given by the probability $2/n(n-1)$ of selecting i and j times p_{ij} the probability of selecting the specific trade. Secondly, when A and B differ by a trade of size one, say between sets A_i and A_j involving vertices k and l, then they also differ by a trade of size one between sets A_k and A_l involving indices i and j (see Lemma 0.2 in main text). Hence, we find $P_{AB} = 2/n(n-1)\,(p_{ij} + p_{kl})$.

To summarize:

$$P_{AB} = \begin{cases} \frac{2}{n(n-1)}\,(p_{ij} + p_{kl}) & \text{if } A \text{ and } B \text{ differ by a trade of size one} \\ \frac{2}{n(n-1)}\,p_{ij} & \text{if } A \text{ and } B \text{ differ by a larger trade} \\ 1 - \sum_{C, C \neq A} P_{AC} & \text{if } A = B, \\ 0 & \text{otherwise.} \end{cases}$$

Normally, the hardest part of proving Theorem AppendixA.1 is showing that the state graph is connected. For the Curveball algorithm we obtain this result for free whenever the state graph of the switch chain is connected: Lemma 0.2 implies that the state graph of the switching model for simple directed graphs is a subgraph of the state graph of the Directed Curveball algorithm because each switch is a trade of size one.

Theorem AppendixA.3. *For any graph G, the Markov chain of the Undirected Curveball algorithm starting at G converges to the uniform distribution on all graphs with the same degree sequences as G.*

Proof AppendixA.3.1. *The state graph of the switching chain for graphs with fixed degree sequence was shown to be connected in [4, 5] which implies the connectance of the state graph of the Undirected Curveball algorithm. The state graph of the Undirected Curveball algorithm is always non-bipartite, since there is a non-zero probability of repeating each state, due to trades of size zero. Finally $P_{AB} = P_{BA}$, since a trade between sets A_i and A_j to form B_i and B_j implies that $|A_{i-j}| = |B_{i-j}|$ and $|A_{j-i}| = |B_{j-i}|$. Hence by Theorem AppendixA.1 the Undirected Curveball algorithm converges to the uniform distribution on its state space.*

AppendixA.2. Uniform sampling Directed Curveball

The transition probabilities of the Directed Curveball algorithm are given by:

$$P_{AB} = \begin{cases} \frac{2}{n(n-1)} p_{ij} & \text{if } B \text{ only differs from } A \text{ in sets } A_i \text{ and } A_j, \\ 1 - \sum_{C, C \neq A} P_{AC} & \text{if } A = B, \\ 0 & \text{otherwise.} \end{cases}$$

Theorem AppendixA.4. *If the state graph corresponding to the switching chain for directed fixed degree sequences is connected, then the Markov chain of the Directed Curveball chain converges to its stationary distribution, which is the uniform distribution.*

Proof AppendixA.4.1. *The state graph of the switching model with respect to directed graphs is a subgraph of the state graph of the Directed Curveball algorithm (see Lemma 0.1 in main text). Hence, a connected state graph of the switching chain implies a connected state graph of the Directed Curveball chain. The state graph of the Directed Curveball chain is always non-bipartite, since there is a non-zero transition probability* P_{AA} *of repeating each state* A *in step (c), due to trades of size zero. Finally* $P_{AB} = P_{BA}$ *for all states* A *and* B *since a trade between sets* A_i *and* A_j *to form* B_i *and* B_j *implies that* $|A_{i-j}| = |B_{i-j}|$ *and* $|A_{j-i}| = |B_{j-i}|$. *Hence convergence to the uniform distribution follows from Theorem AppendixA.1.*

AppendixB. Sampling all simple directed graphs

It is well-known that the switching model for directed graphs can have a disconnected state graph [6]. The simplest example being a directed cycle on three vertices, its opposite orientation can not be obtained by switches, since no switch is possible without introducing self-loops. One approach to ensure all graphs are reached is to introduce an additional move which reorients directed cycles of length three (hexagonal move in [6, 7]. However, we follow another approach that uses a pre-sampling step [8] which is simpler to use in combination with the Directed Curveball algorithm. Furthermore the corresponding Markov chain runs on a (potentially much) smaller state graph which should converge faster.

This approach is based on *induced cycle sets*: an induced cycle set consists of three indices, i_1, i_2 and i_3, for pairs in S such that the vertices $v_{i_1}, v_{i_2}, v_{i_3}$ form a directed cycle in *each* directed graph realisation of S [8]. Berger et al. prove that the state graph of the switching model for degree sequence S is non-connected if and only if S contains an *induced cycle set.* In fact, if S contains k induced cycle sets, then Ψ_S consists of 2^k isomorphic components where each component corresponds to a specific orientation for all k cycles [8]. The same is true for the Directed Curveball algorithm.

Theorem AppendixB.1. *The state graph of the Directed Curveball algorithm decomposes in 2^k isomorphic components, where $k \leq n$ is the number of induced cycle sets. If it is not connected ($k > 0$), then applying the Directed Curveball algorithm on any component leads to the uniform distribution of all states in this component.*

Proof AppendixB.1.1. *It is not hard to see that the state graph of the Directed Curveball algorithm decomposes in the same set of isomorphic components as the switching chain, since any trade is a combination of one or more switches. We have previously shown that the Directed Curveball has a non-bipartite state graph and that $P_{AB} = P_{BA}$. Hence, the Directed Curveball algorithm on a component converges to the uniform distribution on all states in the component.*

This theorem implies that choosing one component uniformly at random and applying the Directed Curveball algorithm on this component leads to a uniform distribution of all states. Hence, we can use the same pre-sampling step as proposed for the switch chain [8] which identifies all induced cycle sets in a directed graph G and chooses a random orientation for each of them. Clearly, using this preprocessing step before starting the Directed Curveball algorithm leads to a uniform sample. We now show that this pre-sampling step can be achieved by the following linear-time algorithm using a result by LaMar [9].

We first define the *corrected Ferrers matrix* for a given degree sequence. Let $S := (a_1, b_1), \dots, (a_n, b_n)$ be a degree sequence in non-increasing lexicographical order. The $n \times n$ *corrected Ferrers matrix* F corresponding to S is a matrix with row sums $b_1, \dots, b_n$. Each row i consists of b_i consecutive 1's followed by consecutive 0's with the exception that the diagonal elements F_{ii} are always 0. This leads to column sums $f_1, \dots, f_n$ of F. The classical result of Chen-Fulkerson-Ryser states that S has a realisation if and only if $\sum_{i=1}^{l}(f_i - a_i) \geq 0$ for all $l \in \{1, \dots, n\}$. For a comprehensive discussion we recommend the paper of Berger [10]. We define $1_x : \mathbb{N} \mapsto \mathbb{N}$ as the function with $1_x(y) = 1$ for $y = x$ and $1_x(y) = 0$ in all other cases.

Theorem AppendixB.2 (Theorem 3.7, [9]). *Let* $S = (a_1, b_1), \ldots, (a_n, b_n)$ *be a lexicographical non-increasing degree sequence with a directed graph as realisation, and* $f_1, \ldots, f_n$ *the column sums of its corrected Ferrers matrix.*
Let $\overline{S} = (b'_1, a'_1), \ldots, (b'_n, a'_n) = (b_{\sigma(1)}, a_{\sigma(1)}), \ldots, (b_{\sigma(n)}, a_{\sigma(n)})$ *be a permutation of* S *which was generated by exchanging the component order in all pairs and sorting it in non-increasing lexicographical order. Let* $f'_1, \ldots, f'_n$ *be the column sums of its corrected Ferrers matrix.*
Indices $i, i+1, i+2$ *form an induced cycle set in* S *if and only if*

1. $(a_i, b_i) = (a_{i+1}, b_{i+1}) = (a_{i+2}, b_{i+2}) = (k, i)$,
2. $(b'_k, a'_k) = (b'_{k+1}, a'_{k+1}) = (b'_{k+2}, a'_{k+1}) = (i, k)$,
3. $\sum_{i=1}^{l} (f_i - a_i) = 1_i(l) + 1_{i+1}(l)$ *for* $l \in \{i-1, \ldots, i+2\}$,
4. $\sum_{i=1}^{l} (f'_i - b'_i) = 1_k(l) + 1_{k+1}(l)$ *for* $l \in \{k-1, \ldots, k+2\}$.

We simplify the above result by showing that items (2) and (4) follow directly from items (1) and (3).

Theorem AppendixB.3. *Let* $S = (a_1, b_1), \ldots, (a_n, b_n)$ *be a lexicographical non-increasing degree sequence with a directed graph as realisation, and* $f_1, \ldots, f_n$ *the column sums of its corrected Ferrers matrix. Indices* $i, i+1, i+2$ *form in* S *an induced cycle set if and only if*

1. $(a_i, b_i) = (a_{i+1}, b_{i+1}) = (a_{i+2}, b_{i+2}) = (k, i)$,
2. $\sum_{j=1}^{l} (f_j - a_j) = 1_i(l) + 1_{i+1}(l)$ *for* $l \in \{i-1, \ldots, i+2\}$,

Proof AppendixB.3.1. *We show that conditions 1.) and 2.) imply that* $i, i+1, i+2$ *is an induced cycle set. We prove that for any adjacency matrix* A *corresponding to a realisation of sequence* S*, these two conditions lead to an induced cycle between vertices* $i, i+1, i+2$*. This shows that each possible realisation possesses such an induced cycle, and hence* $i, i+1, i+2$ *is an induced cycle set.*

Let A *be any adjacency matrix corresponding to a realisation of* S*. Condition 2.) with* $l = i-1$ *states that the number of* $1'$*s in the first* $i-1$ *columns of*

F and A are equal. In other words, the number of $1's$ in all rows from column index 1 to $i-1$ are equal for A and F. Observe that due to the construction of the Ferrers' matrix, the number of $1's$ in a row j of F from index 1 to $i-1$ must always be larger or equal to the number of $1's$ in the same row in A from index 1 to $i-1$. Thus, the sequence of row sums $b_1^{(i-1)}, \dots, b_n^{(i-1)}$ for column indices 1 to $i-1$ must be identical for matrix A and F. (A smaller row sum in A would imply another larger row sum in A). The same is true for the row sums $b_1^{(i+2)}, \dots, b_n^{(i+2)}$ for column indices from 1 to $i+2$ due to condition 2.) with $l = i+2$.

Since $b_i = i$, $b_{i+1} = i$ and $b_{i+2} = i$ by condition 1.), we find $b_i^{(i-1)} = i-1$, $b_{i+1}^{(i-1)} = i-1$, $b_{i+2}^{(i-1)} = i-1$ and $b_i^{(i+2)} = i$, $b_{i+1}^{(i+2)} = i$, $b_{i+2}^{(i+2)} = i$. Hence the $3x3$-sub-matrices of F and A consisting of columns and rows $i, i+1, i+2$ have row sum 1 for each row. Figure B.1 below depicts matrix F.

$$F = \begin{array}{c|cccc|c|c|c|cc|l}
 & 1 & & \dots & & i & i+1 & i+2 & \dots & n & \\
1 & \mathbf{0} & 1 & \dots & 1 & 1 & 1 & 1 & & & \\
\vdots & 1 & \mathbf{0} & \dots & 1 & \vdots & \vdots & \vdots & & & \text{type } (a) \\
 & 1 & 1 & \dots & \mathbf{0} & 1 & 1 & 1 & & & \\
i & 1 & 1 & \dots & 1 & \mathbf{0} & 1 & 0 & & & b_i \\
i+1 & 1 & 1 & \dots & 1 & 1 & \mathbf{0} & 0 & & & b_{i+1} \\
i+2 & 1 & 1 & \dots & 1 & 1 & 0 & \mathbf{0} & & & b_{i+2} \\
\vdots & ? & ? & \dots & ? & 0 & 0 & 0 & & & \\
 & ? & ? & \dots & ? & \vdots & \vdots & \vdots & & & \text{type } (b) \\
n & ? & ? & \dots & ? & 0 & 0 & 0 & & & \\
 & & & & & f_i & f_{i+1} & f_{i+2} & & &
\end{array}$$

Figure B.1: Matrix F.

Combining conditions 2.) and 1.) we find that $f_i = a_i + 1 = k+1$, $f_{i+1} = a_{i+1} = k$, and $f_{i+2} = a_{i+2} - 1 = k-1$. Notice that these conditions imply that for any row l of F with $l \neq i, i+1, i+2$ the values of columns $F_{li}, F_{li+1}, F_{li+2}$ have to equal $1,1,1$ (type (a)) or $0,0,0$ (type (b)). If we allowed a row with $1,1,0$ then there has to be another row $0,0,1$, or two other rows $1,0,1$ and $0,1,1$, neither of which is possible for a Ferrers matrix. The same reason forbids $1,0,0$ as row.

To create a realisation of column i in matrix A we need a 1 less than in

$$
A = \begin{array}{c|cccc|c|c|c|cc|l}
 & 1 & & \dots & & i & i+1 & i+2 & \dots & n & \\
1 & \mathbf{0} & 1 & \dots & 1 & 1 & 1 & 1 & & & \\
\vdots & 1 & \mathbf{0} & \dots & 1 & \vdots & \vdots & \vdots & & & \text{type } (a) \\
 & 1 & 1 & \dots & \mathbf{0} & 1 & 1 & 1 & & & \\
i & 1 & 1 & \dots & 1 & \mathbf{0} & ? & ? & & & b_i \\
i+1 & 1 & 1 & \dots & 1 & ? & \mathbf{0} & ? & & & b_{i+1} \\
i+2 & 1 & 1 & \dots & 1 & ? & ? & \mathbf{0} & & & b_{i+2} \\
\vdots & ? & ? & \dots & ? & 0 & 0 & 0 & & & \\
 & ? & ? & \dots & ? & \vdots & \vdots & \vdots & & & \text{type } (b) \\
n & ? & ? & \dots & ? & 0 & 0 & 0 & & & \\
 & & & & & a_i & a_{i+1} & a_{i+2} & & &
\end{array}
$$

Figure B.2: Matrix A.

column i of F by condition 2.) with $\ell = i$. Let us assume that $A_{\ell,i} = 0$ and $F_{\ell,i} = 1$ with $\ell \neq i, i+1, i+2$. This is only possible when F_ℓ is of type (a). But then we have two different column sums $b_j^{(i+2)}$ in matrices A and F in contradiction to our observation above.

Hence, we can conclude that either a) $\ell = i+1$ or b) $\ell = i+2$ ($\ell = i$ can be excluded because of the demanded diagonal entry 0). For situation a) we find that $A_{i+1,i+2} = 1$ so that A' has row sum 1 for row $i+1$. Furthermore row i has column sum 1 in A' and hence $A_{i,i+1} = 1$ (if $A_{i,i+2} = 1$ then there has to be an index $\ell \neq i, i+1, i+2$ with $A_{l,i+2} = 0$ and $F_{l,i+2} = 1$ which is again a contradiction). Similarly in situation b) we find $A_{i+2,i+1} = 1$ and $A_{i,i+2} = 1$. In both cases A' corresponds to an induced cycle.

Hence all induced cycle sets in a directed graph G can be obtained in linear-time by checking the conditions in Theorem AppendixB.3.

References

[1] L. Lovász. Random walks on graphs: A survey. In *Combinatorics, Paul Erdős is Eighty*, volume 2, pages 353–397. János Bolyai Mathematical Society, 1996.

[2] C. J. Carstens. Proof of uniform sampling of binary matrices with fixed row

sums and column sums for the fast curveball algorithm. *Physical Review E*, 91:042812, 2015.

[3] D. A. Levin, Y. Peres, and E. L. Wilmer. *Markov chains and mixing times.* American Mathematical Society, Providence, Rhode Island, 2009.

[4] R. Taylor. *Combinatorial Mathematics VIII: Proceedings of the Eighth Australian Conference on Combinatoria Mathematics Held at Deakin University, Geelong, Australia, August 25–29, 1980*, chapter Constrained switchings in graphs, pages 314–336. Springer Berlin Heidelberg, Berlin, Heidelberg, 1981.

[5] R. B. Eggleton and D. A. Holton. Simple and multigraphic realizations of degree sequences. In *Combinatorial Mathematics VIII*, pages 155–172. Springer Berlin Heidelberg, 1981.

[6] A. R. Rao, R. Jana, and S. Bandyopadhyay. A Markov chain Monte Carlo method for generating random (0, 1)-matrices with given marginals. *Sankhya: The Indian Journal of Statistics, Series A*, 58:225–242, 1996.

[7] N. D. Verhelst. An efficient MCMC algorithm to sample binary matrices with fixed marginals. *Psychometrika*, 73(4):705–728, 2008.

[8] A. Berger and M. Müller-Hannemann. Uniform sampling of digraphs with a fixed degree sequence. In *Proceedings of the 36th International Conference on Graph-Theoretic Concepts in Computer Science*, pages 220–231. Springer-Verlag, 2010. full version available as Preprint in Arxiv:0912.0685v3.

[9] M. D. LaMar. On uniform sampling simple directed graph realizations of degree sequences. *CoRR*, abs/0912.3834, 2009.

[10] A. Berger. A note on the characterization of digraphic sequences. *Discrete Mathematics*, 314:38 – 41, 2014.
